# Potential Relationship between Poor Oral Hygiene and MRONJ: An Observational Retrospective Study

**DOI:** 10.3390/ijerph20075402

**Published:** 2023-04-05

**Authors:** Silvia D’Agostino, Giulia Valentini, Marco Dolci, Elisabetta Ferrara

**Affiliations:** 1 Complex Unit of Odontostomatology, Interdisciplinary Department of Medicine, University Aldo Moro of Bari, 70121 Bari, Italy; 2 Department of Medical, Oral and Biotechnological Sciences, University Gabriele d’Annunzio, 66100 Chieti, Italy

**Keywords:** MRONJ, osteonecrosis, oral hygiene, non-surgical periodontal therapy

## Abstract

Medication-related osteonecrosis of the jaw (MRONJ) is a drug-related side effect linked but not limited to antiresorptive and antiangiogenic molecules. It recognizes several triggers in dental procedures, such as surgery, endodontic treatments, and root planing, but also prosthesis decubitus or with a spontaneous onset. Although there are many reports about the onset of this pathology, oral hygiene status is mainly described as a consequence of MRONJ. Not so much is known about the oral hygiene situation as a concurrent factor in the pathogenesis of severe stages and about non-surgical periodontal therapy in patients affected by MRONJ. Actually, clear instructions for non-surgical periodontal therapy are poor in the literature. The primary outcome of the present study is to evaluate the oral hygiene status in MRONJ patients. In addition, a secondary outcome is to review the factor of poor oral hygiene as a cause or worsening aspect for MRONJ. A total of 45 subjects (19 males and 26 females) with a mean age of 59 ± 12 were enrolled. The Pearson correlation coefficient showed no significant results for the variable of the Simplified Oral Hygiene Index (OHI-S) and the American Association of Oral and Maxillofacial Surgeons (AAOMS) stage, although the majority of patients showed poor oral hygiene with an OHI-S average of 3.39 ± 1.83. As stated by the last AAOMS position paper, poor plaque control is related to a worsened MRONJ stage. The relation between the lack of oral hygiene and MRONJ onset is still unclear.

## 1. Introduction

Bone impairment is a direct consequence of several conditions (osteoporosis, secondary localization of tumors, osteogenesis imperfecta, and Paget’s disease), causing fractures and bone or articular pain, and subsequently playing a relevant role in lowering Quality of Life (QoL). Many efforts have been made in the past to prevent the aforementioned disabling complication and pharmacological treatments have been proposed to minimize the impact of these pathologies in reducing bone loss. Medications active toward bone resorption and secondary lesions treatment were proposed to enhance QoL, although the risk to develop medication-related osteonecrosis of the jaw (MRONJ). MRONJ is a drug-related side effect that recognizes a trigger in dental procedures. The first definition of this pathology was related to bisphosphonates (BPs) use in 2007 and called bisphosphonates-related osteonecrosis of the jaw (BRONJ) [1]. Due to the evidence that it could also be related to other drugs, it has been changed during the years with the more comprehensive MRONJ, described by the American Association Of Oral and Maxillofacial Surgeons (AAOMFS) as “current or previous treatment with antiresorptive or antiangiogenic agents; (1) exposed bone or bone that can be probed through an intraoral or extraoral fistula(e) in the maxillofacial region that has persisted for more than 8 weeks; and (2) no history of radiation therapy to the jaws or obvious metastatic disease to the jaws” [2,3,4,5].

Khan et al. (2015) found that the incidence in the osteoporotic population is very low, ranging from 0.001% to 0.15% person–years of exposures, barely higher than in the general population. On the other hand, in the oncologic population the incidence seemed to be related to many factors, including the time and type of therapy adopted [4]. In the last years, many dental conditions and procedures were advocated as triggers of this complication, most of them related to tooth extraction and oral surgery procedures, endodontic treatment, and periodontal therapies [6,7,8,9,10,11]. A paper by Ruggiero et al. noted that the majority of ONJ cases in oncologic patients treated with BP had previous dental procedures, although the mechanisms of this association were unclear. In this paper, endodontic treatment was suggested as a low-risk procedure eligible for residual roots to avoid extractions [6]. Endodontic treatment, however, has been pointed out as a trigger in ONJ developing, both for primary treatments and endodontic failures. This second hypothesis seems to be supported by the presence of residual local inflammatory conditions established by endodontic treatment failure or mishandling such as root perforation, root canal overfilling/underfilling, and root fracture [7]. Some authors recently concluded that periodontal disease seems to be advocated as a low-grade inflammatory local disease, representing a possible trigger for MRONJ; however, further studies should be performed to clearly state this hypothesis [8,12]. Dental extraction and other surgical procedures have been related to the development of such an invalidating condition [9,10]. Although there are many reports about the onset of this pathology, oral hygiene status is mainly linked as a consequence of MRONJ; not so much is known on the oral hygiene situation as a concurrent factor in the pathogenesis of severe stages and on non-surgical periodontal therapy in patients affected by MRONJ. The last AAOMS position paper (2022) clearly states that systemic inflammation is implicated in pathogenesis and dental hygiene procedures seem to be neglected in MRONJ patients, while highly recommended [13]. In the first Position Paper, AAOMS stated that “Maintaining good oral hygiene and dental care is of paramount importance in preventing dental disease that may require dentoalveolar surgery” [1]. Periodontopathogenic bacteria, such as *Fusobacterium nucleatum*, associated with pamidronate may play a crucial role in delaying the gingival mucosa healing and subsequently produce a BRONJ-like lesion [14]. Although bacteria and infections seem to contribute, their role is not clearly defined in the literature; in other words, there is no evidence if infection follows or precedes bone necrosis [4]. In this light, oral hygiene is a key point in prevention, but the same value is not assessed for treatments [15]. Poxleitner et al. underline that the number of patients exposed to antiresorptive and antiangiogenetic treatments will increase in the future, and prevention will be a core question in QoL assessments [16]. Clear instructions for non-surgical periodontal therapy are poor in the literature; Khan et al. in 2015 referred to this, but without an accurate protocol [4]. Pittman et al. underline that careful oral hygiene should be established in association with other therapies, both conservative and surgical treatments [17]. According to Nicolatou-Galitis et al., dental hygiene procedures are part of the best dental practices in low- and high-risk patients and should be carefully executed [5]. Vandone et al. in 2012 observed that after establishing preventive oral care measures, a lower number of cases of ONJ were observed [18], and Krimmel et al. observed that drugs were not the only cause, but oral and dental health were crucial elements in the onset of this pathology [19,20]. Dental evaluation is evidently essential to avoid MRONJ, especially because the drugs involved in this disease are often the first therapeutical option for metabolic and oncologic patients, mostly keeping in mind the moderate incidence of this side effect [21]. At the same time, a paper by Al Abdullateef et al. suggested that careful information about this kind of complication should be given both to dentists and patients, to avoid or delay the onset of complications [22]. The primary outcome of the present study is to evaluate the oral hygiene status in MRONJ patients. In addition, a secondary outcome is to propose a protocol for non-surgical periodontal treatment in MRONJ patients.

## 2. Materials and Methods

### 2.1. Study Design

**Figure 1:**
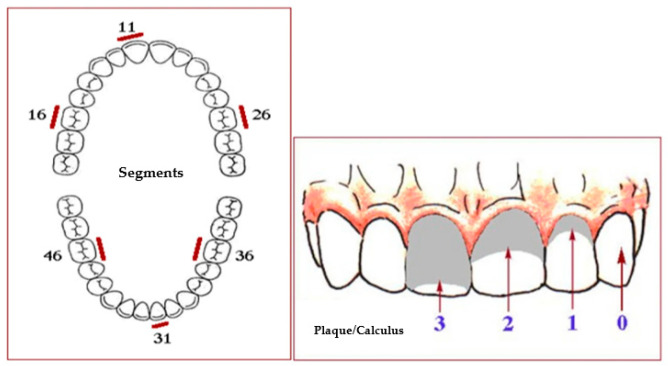
OHI-S scheme. Fregatto LF et al. 2021 (modified).

An observational study was conducted between July 2015 and October 2022 to investigate the potential association between poor oral hygiene and MRONJ. Forty-five outpatients presenting clinical or radiographic evidence for MRONJ were identified according to the criteria defined by the American Association of Oral and Maxillofacial Surgeons 2014 (AAOMS) [3] and were consecutively enrolled. The data were collected through personal interviews and clinical examinations. The demographic data collection included age and gender. On the same day of enrolment, a detailed oral hygiene test was assessed by employing the Simplified Oral Hygiene Index (OHI-S) [23]. The OHI-S is a quantitative expression of oral cleanliness, composed of the combined “Debris Index” and “Calculus Index-Simplified.” The indices are based on 12 numerical recordings of three segments of each dental arch: (1) the segment distal to the right cuspid, (2) the segment distal to the left cuspid, and (3) the segment mesial to the right and left first bicuspids. Each determination indicates the amounts of debris or calculus on the buccal and lingual surface [24] (Figure 1).

Both indices are calculated separately and are added to obtain the OHI-S score for an individual. OHI-S scores may be interpreted as: good (0–1.2), fair (1.3–3.0), and poor (3.1–6.0). The patients were examined employing sterile mouth mirrors and a probing depth probe (UNC 15 periodontal probe; Hu-Friedy Mfg. Co., Chicago, IL, USA) in accordance with the recommendations of the World Health Organization [25]. All clinical examinations were performed by the same two operators. This research complies with the principles of the Helsinki Declaration. Written informed consent was obtained from patients and healthy subjects before enrolling in the trial. The protocol was approved by the local ethics committee of the researchers’ universities.

### 2.2. Statistical Analysis

The collected data were analyzed using Statistical Package for Social Statistics version 20.0 (IBM SPSS Statistics 20, Chicago, IL, USA). Descriptive statistics such as mean ± SD were used to describe the results as continuous variables. In addition, the frequency and percentage of the categorical variables were reported. To evaluate the association between the stage of MRONJ and oral hygiene, a Pearson’s correlation analysis was conducted between AAOMS Stage and OHI-S. Cohen’s standard was used to evaluate the strength of the relationship, where coefficients between 0.10 and 0.29 represent a small effect size, coefficients between 0.30 and 0.49 represent a moderate effect size, and coefficients above 0.50 indicate a large effect size. A value of *p* of ≤ 0.05 was considered to be statistically significant.

## 3. Results

### Assumptions 

**Figure 2:**
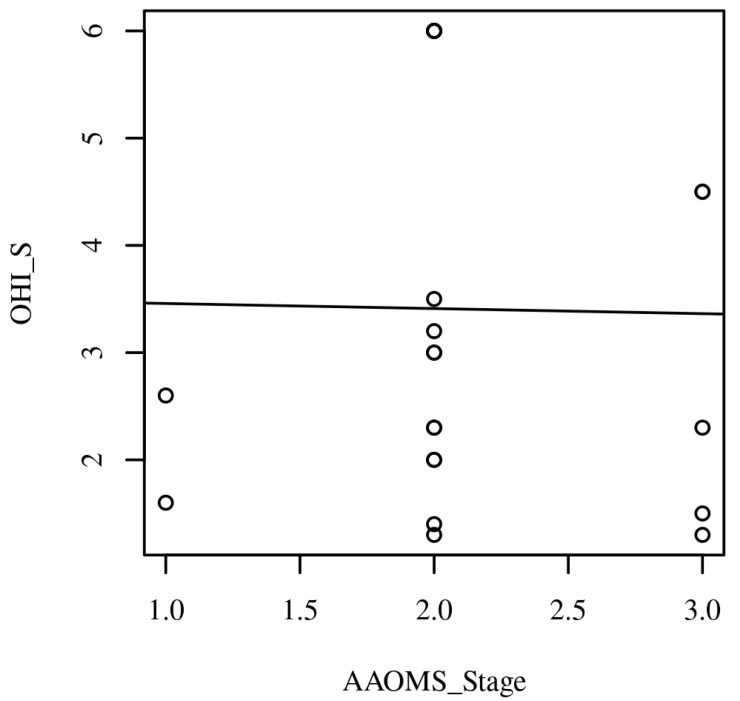
Scatterplots with the regression line added for AAOMS Stage and OHI-S.

*Linearity*. A Pearson’s correlation requires that the relationship between each pair of variables is linear. Figure 1 presents the scatterplot of the correlation. A regression line has been added to percentages which were calculated for each nominal and ordinal variable (Figure 2).

Descriptive analysis was showed in Table 1.

**Figure 3:**
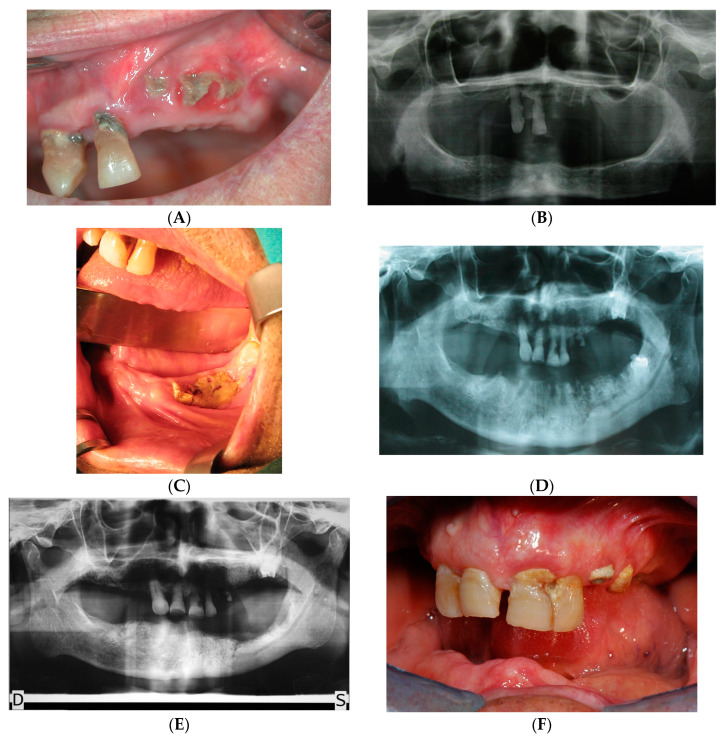
Clinical and radiological examinations. (**A**) Female patient 89 years old Stage 1 AAOMS. Necrotic exposed bone in the upper left jaw with poor oral hygiene status. (**B**) Orthopantomogram of the same patient. (**C**) Male patient 80 years old. Stage 1 AAOMS. (**D**) Orthopantomogram of the same patient. (**E**) Orthopantomogram after surgical ablation. D, right. S, left. (**F**) Post-surgical plaque control remained unsatisfactory.

A total of 45 subjects (19 males and 26 females) between the ages of 48 to 76 with a mean age of 59 ± 12 were enrolled. As shown in Table 1, the most frequently observed category of Localization of lesions was Upper jaw (*n* = 21, 46.67%). The most frequently observed category of AAOMS_Stage_1 was (1, 2] (*n* = 31, 68.89%) (Figure 2). The most frequently observed category of Drug was zoledronic acid (*n* = 32, 71.11%). The most frequently observed category of Sex was F (*n* = 26, 57.78%). The frequencies and percentages are presented in Table 1. The majority of patients showed poor oral hygiene: the observations for OHI-S had an average of 3.39 (SD = 1.83, SEM = 0.27, Min = 1.10, Max = 6.00, Skewness = 0.27, and Kurtosis = −1.48). The observations for AAOMS Stage had an average of 2.13 (SD = 0.55, SEM = 0.11, Min = 1.00, Max = 3.00, Skewness = 0.10, and Kurtosis = 0.23). The summary statistics can be found in Table 2. Examples of patients included are shown in Figure 3.

The result of the correlation was examined based on an alpha value of 0.05. There were no significant correlations between any pairs of variables. Table 3 presents the results of the correlation.

## 4. Discussion

Extensive efforts have been dedicated to identifying a potential cause for the development of MRONJ, and the association between local factors and this severe debilitating condition has been extensively studied. Inadequate oral hygiene is one of the elements that affect the occurrence of MRONJ, although it has not been found to be a key driver. In this study, we aimed to examine the correlation of oral hygiene status and osteonecrosis of the jaws investigating a cohort of patients with diagnosed ONJ. The predictors of the occurrence and severity of MRONJ have been the subject of previous research, with a higher risk of MRONJ in patients with specific comorbidities and/or local factors in-volving tooth extraction, periapical, and periodontal infections both with and without tooth extraction, poor oral hygiene, and inadequate prostheses. Renal dialysis, erythropoietin therapy, diabetes, and hypothyroidism have been found to be associated with an increased risk for promoting MRONJ. One factor that links these groups and is consistently observed is lower bone mineral density (BMD) and the impaired vascular system, resulting in a reduced blood supply to the bone structures, particularly the cortical structures such as the lower jaw. The relative importance of oral hygiene on the risk of developing this complication is often cited as a fundamental aspect for the prevention of infections in patients undergoing drug treatments with risk of osteonecrosis. Poor oral hygiene conditions would result in several opportunistic infections. It has been demonstrated that patients with scarce oral hygiene are a subset of people with an increased likelihood of developing this complication. The Marx et al. [26] study evaluated the relation between bacterial plaque amount and MRONJ and demonstrated that it was linked with the occurrence of osteonecrosis. In addition, the authors revealed a linear pattern regarding the manifestation of periodontal disease, strictly related to bacterial plaque accumulation, in relation to the diagnosis of MRONJ in 84% of patients. Walter et al. [27] also reported that BRONJ patients had a higher prevalence of calculus and visible plaque (87.5%) compared with patients undergoing BPs administration and no signs of osteonecrosis (68%). The present study found no strong statistical evidence to support the existing literature claiming an association between oral hygiene status and MRONJ. Differently, but in line with the findings of the present study, Carmagnola et al. [28] reported that oral hygiene had no role in ONJ occurrence. In this study, although the estimates for poor oral hygiene on the risk of MRONJ were not entirely consistent, it is likely that scarce oral conditions increased the risk of oral infection, with more possible convincing estimates for periodontal patients. Our analysis did not reveal a significant correlation between oral hygiene status and MRONJ, presumably because of the small sample size and un-representative population; nevertheless, the causes are consistent across populations. If more cases had been included in the study, the likelihood of identifying a demonstrable association could have been increased. In addition, the presence of poor oral hygiene could not entirely explain the onset of the pathological condition. However, maintaining good oral hygiene has a higher effect on reducing the risk of infection, directly related with the occurrence of MRONJ. Contrary, Kuchur et al. [29] demonstrated that patients in a treatment with BPs and manifesting BRONJ had a poor standard of oral health compared to patients under BPs without BRONJ (had poorer oral hygiene, more complications of caries, and a worse periodontal status). All of the studies agreed about the importance of good oral hygiene to prevent complications due to dental plaque and periodontal disease, without suggesting a clear and univocal non-surgical periodontal treatment protocol applied to every stage of the disease. MRONJ is a complex disease with features that complicate the detection of the disease’s contributing factors, and the effect of oral hygiene status may be obscured or confounded by other contributing factors.

Based on the last AAOMS position paper [13], five categories of patients may be identified.

*Patient at-risk*. Asymptomatic patients who have been treated with intravenous (IV) or oral antiresorptive therapy without apparent necrotic bone need to be assigned to accurate trimestral scaling. Eventual root planing (RP) has to be performed following detailed informed consent about the risk of ONJ in this procedure [30]. Photobiomodulation (PBM) with Low-Level Laser Therapy (LLLT) should be performed to improve the healing of soft tissues [31,32,33].

*Stage 0*. Nonspecific symptomatic patients with no clinical evidence of necrotic bone should be strictly supervised for their oral hygiene status, trimestral scaling is mandatory, and it may require closer recall sessions tailored to the patient’s skills to maintain good plaque control. RP should be scheduled only in stages III and IV of the New Periodontal Classification (NPC) [34] following detailed informed consent. PBM through LLLT should be performed to improve the recovery of the subgingival treated sites. Chlorhexidine (CHX) mouthwashes 0.12% and CHX gel 1% have been suggested by some authors [35].

*Stage 1*. Asymptomatic patients with exposed necrotic bone and/or fistula probing to the bone without inflammation/infection need to be assigned to individualized scaling sessions following their own aptitude to home oral hygiene procedures and general conditions. The dental practitioner should not fear to properly perform the scaling of teeth included in the necrotic area; otherwise, they would be considered as microbial reservoirs. An adequate RP of the vital element in stages III and IV NPC is recommended. LLLT of both necrotic and vital sites may be accomplished [36,37]. A CHX mouth rinse and gel as in the previous stage 0 are advised.

*Stage 2 and 3*. Symptomatic patients with exposed necrotic bone and/or fistula probing to the bone with inflammation/infection and/or extension of the necrosis to the basal bone or contiguous structures (maxillary sinus, extraoral fistula, and pathological fractures) need to be allocated to scaling sessions after antibiotic administration following their general condition. RP, LLLT, and CHX mouthwashes and gels may be carried out. In this case, the recall program needs to consider the future therapies the patients will face and the multidisciplinary approach is mandatory [38].

### Limitations of the Study

The methodological issues need to be noted. This is an observational study with an improvable sample size; in order to gather more complete information about the oral hygiene status of MRONJ patients and in order to find out a possible correlation, the authors aim to amplify the sample size and enroll more patients. Finally, the observation period was too short to draw statistically significant conclusions; a longer follow-up is needed to obtain more information.

## 5. Conclusions

The oral biofilm control should be mandatory for MRONJ patients as it is a worsening factor in more severe AAOMS classification stages; pus and infection are elements accountable in stages 2 and 3. Professional dental hygiene is often indicated in the last position papers but without a clear and univocal operative protocol for practitioners who have to face these patients. 

## Figures and Tables

**Table 1:** Frequency Table for Nominal and Ordinal Variables.

Variable	*n*	*%*
*Localization*		
Upper jaw + lower jaw	10	22.22
Upper jaw	21	46.67
Lower jaw	14	31.11
*AAOMS_Stage_1*		
(−Inf, 1]	3	6.67
(1, 2]	31	68.89
(2, 3]	11	24.44
(3, Inf]	0	0.00
*Drug*		
Zoledronic acid	32	71.11
+Alendronic acid	1	2.22
Alendronic acid	6	13.33
Monoclonal antibodies	6	12.11

**Table 2:** Summary statistics.

Variable	M	SD	SEM	Min	Max	Skewness	Kurtosis
OHI-S	3.39	1.83	0.27	1.10	6.00	0.27	−1.48
AAOMS_Stage	2.13	0.55	0.11	1.00	3.00	0.10	0.23

**Table 3:** Pearson’s correlation results between AAOMS Stage and OHI-S.

Combination	*r*	95.00% CI	*p*
AAOMS_Stage-OHI-S	−0.01	[−0.42, 0.40]	0.948
